# Impact of *Pseudomonas aeruginosa* quorum sensing signaling molecules on adhesion and inflammatory markers in endothelial cells

**DOI:** 10.3762/bjoc.14.235

**Published:** 2018-10-05

**Authors:** Carmen Curutiu, Florin Iordache, Veronica Lazar, Aurelia Magdalena Pisoschi, Aneta Pop, Mariana Carmen Chifiriuc, Alina Maria Hoban

**Affiliations:** 1 University of Bucharest, Faculty of Biology, Department of Microbiology-Immunology, Bucharest, Romania; 2 Research Institute of the University of Bucharest, Romania; 3 University of Agronomical Sciences and Veterinary Medicine, Faculty of Veterinary Medicine, Bucharest, Romania; 4 Institute of Cellular Biology and Pathology Nicolae Simionescu of Romanian Academy, Romania

**Keywords:** adhesion, host–pathogen interaction, inflammation, Pseudomonas, quorum sensing

## Abstract

*Pseudomonas aeruginosa* relies on the quorum sensing (QS) signaling system as a central regulator mechanism of virulence expression that contributes to the formation and maintenance of biofilms and tolerance to conventional antimicrobials. QS Signaling molecules (QSSMs) may be recognized and may function also within the host cells, being potentially involved in the progression of the infectious process. In this study we evaluate the expression of adhesion and inflammatory molecules in endothelial cells treated with *P. aeruginosa* QSSMs, in order to bring new insights on the mechanisms involved in the interaction of *P. aeruginosa* with host cells during the infectious process. Endothelial cells were stimulated with 20 µM of main *P. aeruginosa* QSSMs (OdDHL = *N*-(3-oxododecanoyl)-L-homoserine lactone, C4HSL = *N*-butyryl-L-homoserine lactone, PQS = 2-heptyl-3-hydroxy-4(1*H*)-quinolone and HHQ = 2-heptyl-4-quinolone). Adherence to endothelial cells, inert substratum and biofilm formation was evaluated. The expression of adhesion molecules (VE-cadherin, PECAM-1, ICAM-1, and P-selectin) and inflammatory response molecules (IL-1β, IL-6, TNFα, TGFβ, and eNOS) was assessed by qRT-PCR and flow cytometry. Our results showed that bacterial adherence to inert substratum and biofilm were decreased in the presence of all tested QSSMs. The adherence index of PAO1 laboratory strain to host cells was decreased between 10–40% in the presence of QSSMs, as compared to untreated control. Expression of eukaryotic cells adhesion molecules ICAM-1 and P-selectin was stimulated by QSSMs, whereas VE-cadherin and PECAM-1 levels were increased only by C4HSL. The inflammatory response of endothelial cells was also modulated, as observed by the modified expression of IL-1β (for C4HSL, PQS and HHQ), IL-6 (for C4HSL and HHQ), TNFα (for C4HSL and HHQ), TGFβ, and eNOS factors. Our results demonstrate that the main pseudomonadal QSSMs differentially modulate endothelial cells adhesion and proinflammatory cytokine expression. These observations provide new insights in the mechanisms by which different QSSMs activate endothelial cells and modulate the infectious process, and support the importance of recent studies aiming to develop anti-QS therapeutic strategies to fight against *P. aeruginosa* infections.

## Introduction

*Pseudomonas (P.) aeruginosa* is an opportunistic pathogen that causes severe and persistent infections in immune compromised individuals and in patients with bronchiectasis or cystic fibrosis. The infections become chronic, as *P. aeruginosa* develops resistance to conventional antibiotics due to its ability to produce virulence factors and modulate immune defenses by *quorum sensing* (QS) and biofilm production. *Pseudomonas aeruginosa* is recognized as the principal pathogen responsible of high morbidity and mortality in patients with cystic fibrosis, one of the most common life-threatening autosomal recessive genetic disease in Northwest European populations, determined by mutations in the cystic fibrosis transmembrane conductance regulator (CFTR) gene [1]. This mutation determines alteration of ion transport and subsequent dehydration of the airway surface liquid, resulting in a viscous mucus layer on the airway surface of cystic fibrosis patients that deteriorate the mucociliary clearance and enhance the infection, inflammation and respiratory insufficiency [2]. *P. aeruginosa* pathogenesis is multifactorial, as suggested by the large number and wide spectrum of bacterial virulence factors present either attached to the cell wall or extracellular virulence factors capable of producing massive tissue damage and blood dissemination of the infection [3]. The regulation of the virulence factors expression is coordinated by quorum sensing (QS), an intercellular communication system based on cell density dependent molecules with autoinductory properties that play a pivotal role in the pathogenesis of various infections. *P. aeruginosa* produce two types of quorum-sensing signaling molecules (QSSMs): *N*-acylhomoserine lactones (AHL) and 2-alkyl-4-quinolone (PQS) derivatives. The AHLs molecules described so far in *P. aeruginosa* belong at two quorum sensing (QS) systems: *las* and *rhl* systems whose autoinducer (AI) molecules are *N*-(3-oxododecanoyl)homoserine lactone (OdDHL, 3-oxo-C12-HSL), and *N*-butyryl-L-homoserine lactone (C4-HSL), respectively. AHL systems are interconnected by a third mechanism that uses signaling molecules such as 2-alkyl-4-quinolone (AQ), the most relevant one being 3-hydroxy-4-quinolone (PQS = Pseudomonas quinolone signal) and its immediate precursor 2-heptyl-4-quinolone (HHQ) [4–5]. QS systems modulate numerous microbial virulence features, such as bacterial adherence and biofilm formation. It was proved that QS signaling plays an important role in cell attachment, in the differentiation of Pseudomonas biofilms and even biofilm survival [6]. The *las* system is indispensable for the development of a normal biofilm, including the differentiation of biofilm-like structures (such as “mushrooms” and "columns") observed in biofilms developed in vitro [7]. Some studies have shown that QS-deficient mutant strains initially form biofilms with the same dense structure as wild strains. The mutant strains produce even "mushroom"-like structures similar to wild strains that have as carbon source, glucose, but compared to wild type, mature biofilms (10 or more days of development) produced by QS deficient strains differ in size and stability of the structure, being more flexible due to the production of QS-regulated extracellular DNA [8], which acts as a stabilizer of three-dimensional biofilm structure [8]. In patients with cystic fibrosis, the growth of bacteria in biofilm determines aggravation of the disease. Recent studies have found that the QS molecules interact with eukaryotic cells and modulate also host immune response [9]. But, although the roles of the *N*-acylhomoserine lactones in the modulation of immune response have been well studied, the results obtained until now were contradictory, depending on the cell line, the concentration and the time of action [10]. Moreover, the effects of AQs and other Pseudomonas molecules on the modulation of immune responses are poorly understood. The elucidation of the intimate QSSM mechanisms could contribute to the development of new anti-QS therapeutic strategies against *P. aeruginosa* infections, which are very difficult to treat.

The vascular endothelium is crucial for cell and tissue homeostasis and regulation of inflammatory response. The loss of its integrity causes plasma, proteins and cells to build up in the interstitial space, resulting in inflammation [11]. In order to avoid both chronic and acute inflammatory disease, e.g., atherosclerosis or loss of vascular volume, such as in septic shock, the soundness of the vascular permeability barrier is essential. The barrier function of the endothelium is strictly controlled by intercellular adherence junctions (AJ) and tight junctions (TJ), interconnected with cytoskeletal proteins. 3O-C12-HSL induces breaks in the epithelial barrier, disrupting cell junction and enhanced permeability by alterations in the phosphorylation status of TJ and AJ proteins [12]. The transmembrane protein vascular endothelial cadherin (VE-cadherin) is the major structural component of endothelial AJ with a pivotal role in endothelial barrier integrity. LasB protease released by *P. aeruginosa* during infection determines VE-cadherin cleavage and facilitates type III secretion system toxicity in endothelial cells [13]. ICAM-1 (intercellular adhesion molecule 1) and PECAM-1 (platelet endothelial cell adhesion molecule 1) are endothelial- and leukocyte-associated transmembrane proteins that permit transmigration of leukocytes into tissues and are induced by interleukins (IL-1, IL-8), tumor necrosis factor (TNF), nitric oxide (NO), and other inflammatory and stress factors. The investigations of the role of QSSM produced by *P. aeruginosa* on vascular endothelial cells (EC) are poorly investigated. The aim of this study was to evaluate the expression of adhesion and inflammatory molecules in endothelial cells treated with *P. aeruginosa* quorum-sensing (QS) molecules, in order to elucidate their role in the occurrence of tissue damages, in which endothelial cells are involved, such as: wound healing, transepithelial migration of neutrophils, lung inflammation and permeability. Also, we highlighted the impact of QSSMs on microbial attachment at inert and cellular substrata and biofilm formation in vitro.

## Results and Discussion

### Adherence and biofilm development

**Figure 1:**
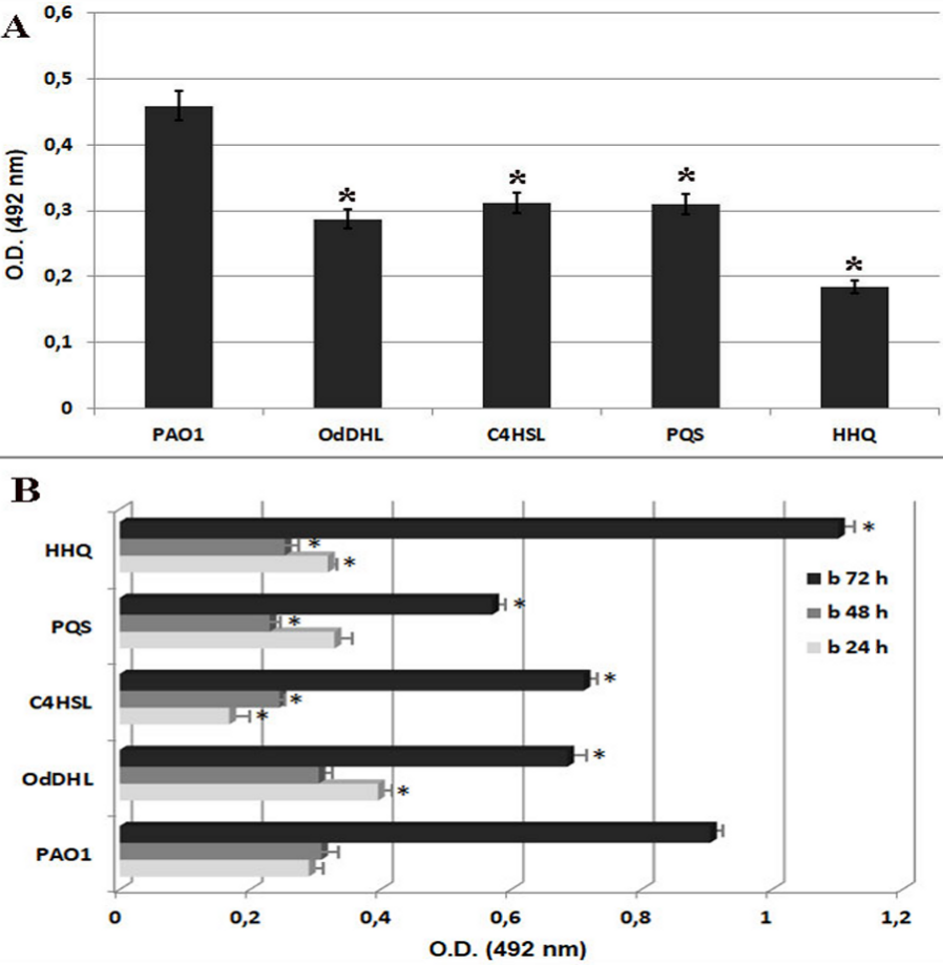
Graphic representation of the bacterial adherence to inert substrata (A) and biofilms developed after 24 h, 48 h and 72 h of incubation (B) in the presence of 20 µM purified QSSMs (*P < 0.05, based on ANOVA and Bonferroni post test of medians of 3 independent experiments performed in triplicate).

Even though there are numerous recent studies about the influence of bacteria QSSMs on host cells, their results are chiefly about the effects of AHL molecules, whereas the interactions between host cells and PQS have remained largely unknown. As far as we know, this is the first publication showing how both *P. aeruginosa*-derived AHL and PQS impact adhesion and inflammatory parameters of endothelial cells. Also, the impact of the main *P. aeruginosa* QSSMs on key bacterial virulence factors such as attachment and biofilm formation was shown, as these behaviors are very important for the progression of the infectious process and represent the main host-pathogen interactions. Bacterial adherence to inert substrata and biofilms developed at different periods of time (24 h, 48 h and 72 h) were decreased in the presence of all tested QSSMs in PAO1 culture (Figure 1). Adherence to inert substratum was decreased with 37.4% for OdDHL, 32.2% for C4HSL, 32.4% for PQS and with 60% for HHQ compared with PAO1 control (Figure 1a). Biofilm formation was slightly inhibited by the presence of most of the tested OSSMs; only HHQ slightly increased the development of biofilm (21.7%) at 72 hours (Figure 1b).

**Figure 2:**
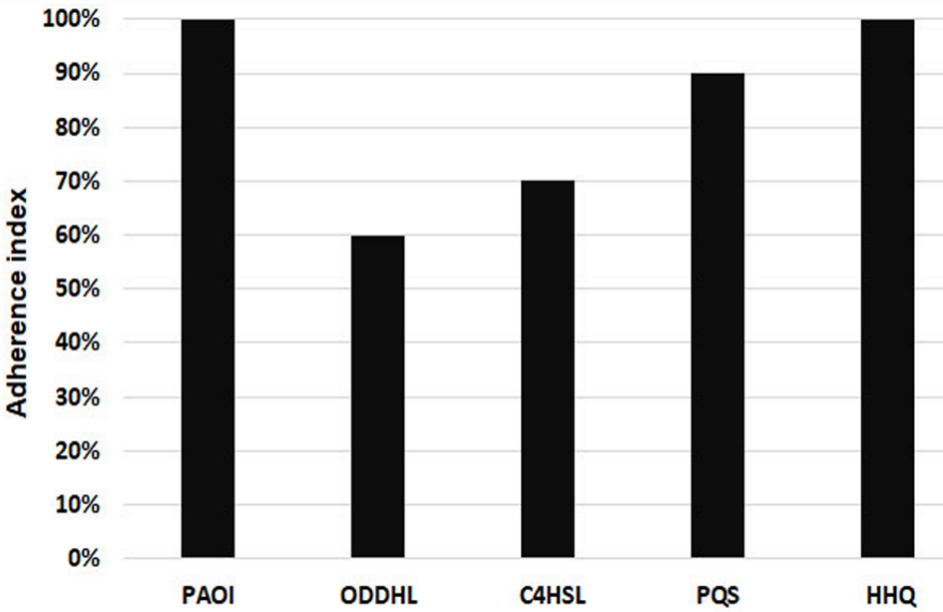
Graphic representation of the adherence index to cellular substrata of PAO1 strain treated with 20 µM of tested purified QSSMs.

**Figure 3:**
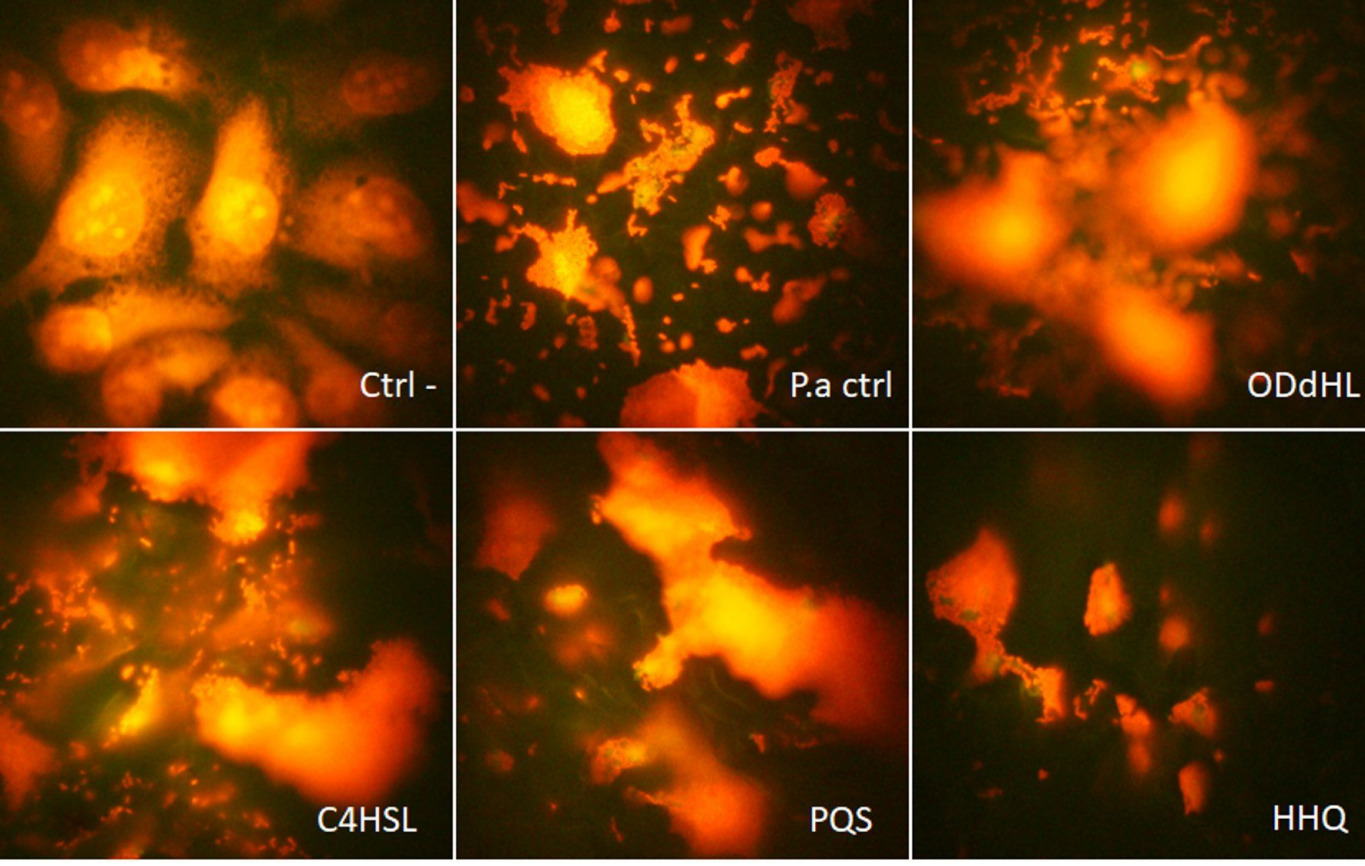
Fluorescence microscopy images of QSSMs treated euckariote cells, revealing attachment patterns. (Ctrl- = untreated control cells, P.a ctrl = *P. aeruginosa* control treated cells, ODdHL = *P. aeruginosa* + OddHL treated cells, C4HSL = *P. aeruginosa* + C4HSL treated cells, PQS = *P. aeruginosa* + PQS treated cells, HHQ = *P. aeruginosa* + HHQ treated cells, ob. 100×, immersion oil.

The inhibitory effect of adherence was also observed for cellular substrate adherence. The adherence index of PAO1 strain to endothelial cells was decreased in the presence of 20 µM QSSMs compared to untreated control. The inhibitory effect was observed in the case of OdDHL (40% inhibition) and C4HSL (30% inhibition), and also a low inhibition (10%) was observed in the case of PQS (Figure 2). HHQ had no significant effect on the adherence index of *P. aeruginosa* to host endothelial cells, but a change in the adherence pattern of bacteria to the endothelial cells was registered, from aggregative (control PAO1) to diffuse aggregative. As seen in Table 1, changes in the adherence pattern were observed in the presence of all tested QSSMs. The main tendency of the tested QSSMs is to disperse microbial aggregates attached on the host cells, since it is clearly seen that the untreated PAO1 control has a higher aggregative potential, as compared to all other samples (Figure 3).

**Table 1:** The adherence *patterns* to cellular substrate of PAO1 strain treated with 20 µM purified QQSMs.

sample	adherence pattern

control PAO1	aggregative
OdDHL	diffuse aggregative
C4HSL	localized aggregative
PQS	diffuse aggregative
HHQ	diffuse aggregative

**Figure 4:**
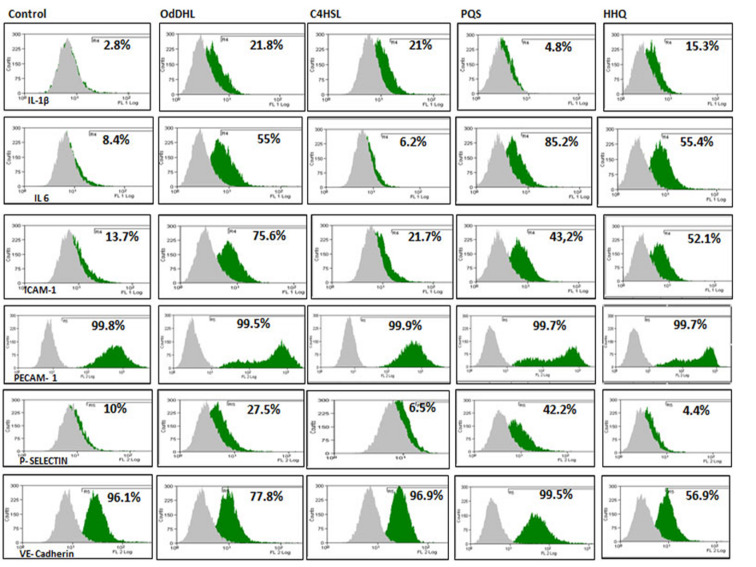
Flow cytometry assays for inflammatory (IL-1β, IL-6), and adhesion markers (ICAM-1, PECAM-1, P-selectin and VE-cadherin) in endothelial cells treated with PAO1 and QSSMs (*P < 0.05, Bonferroni post test of medians of 3 independent experiments performed in triplicate (*n* = 3)).

Flow cytometry analysis of endothelial cells adhesion molecules revealed that ICAM-1 and P-selectin production increased for all QSSMs treated samples, as compared with control cells, the highest effect being observed in case of OdDHL and PQS. ICAM-1 showed an increased expression in endothelial cells treated with OdDHL (75.6% ± 0.48), C4HSL (21.7% ± 0.04), PQS (43.2% ± 0.011), HHQ (52.1 ± 0.019), as compared to control cells (13.7% ± 0.50). P-selectin expression was also increased for OdDHL (27.5% ± 0.03) and PQS (42.2% ± 0.04) treated cells, while C4HSL (6.5% ± 0.28) and HHQ (4.4% ± 0.02) treated samples provided lower values, comparable to untreated control cells (10% ± 0.19). The expression of PECAM-1 and VE-cadherin, were strongly positive for all QSSMs treated cells, as they are in untreated control endothelial cells (Figure 4). These results suggest an inflammatory/reactive response of endothelial cells, explained by the stimulation of ICAM-1 and P-selectin expression which correlate with an increased production of inflammatory cytokines (such as IL-1, IL-8, TNFα). These data are confirmed by recent literature showing the immunomodulatory effects of OdDHL and PQS signaling molecules [14]. It has been shown that OdDHL determines the overexpression of mRNA for IL-8 and stimulates the production of numerous active cytokines in fibroblasts and human epithelial cells in vitro. High concentrations (100 μM) of purified OdDHL activate the p42/44 MAPK signaling pathway and subsequently the NFκB transcription factor that stimulates the production of IL-8 proinflammatory cytokines [15] in human lung or fibroblast bronchial cells. 3-Oxo-C12-AHL also activates extracellular signal-regulated kinases (ERKs), which subsequently induces the activation of NFκB transcription factor. Thus, activation of NFκB is essential for maximal IL-8 production following stimulation of 3-oxo-C12-AHL cells [16]. In addition, the same group later demonstrated that *P. aeruginosa* can induce stimulation of prostaglandin E2 (PGE2) production via the COX-2 cyclooxygenase pathway. In vitro studies on human lung fibroblasts have shown that OdDHL causes overexpression of *cox-2* but not *cox-1*, and this effect appears to be related to NFκB activation. These autoinducers (AIs) stimulate the production of membrane-associated prostaglandin E (PGE) and PGE2 but not PGE from cytosol. It is known that PGE2 plays a role in inducing mucus secretion, vasodilatation and edema, acting as a lipid mediator in immunomodulation [17]. These results indicate that OdDHL contributes to the induction of inflammation and pulmonary pathology in *P. aeruginosa* infections, which is mainly visible in cystic fibrosis patients. PGE2 and COX-2 have been shown to play an important role in suppressing the production of reactive oxygen species by diminishing the bacterial clearance process by macrophages. For these reasons, both PGE2 and COX-2 have been proposed as future therapeutic targets for the treatment of severe pneumonia produced by *P. aeruginosa* [18].

**Figure 5:**
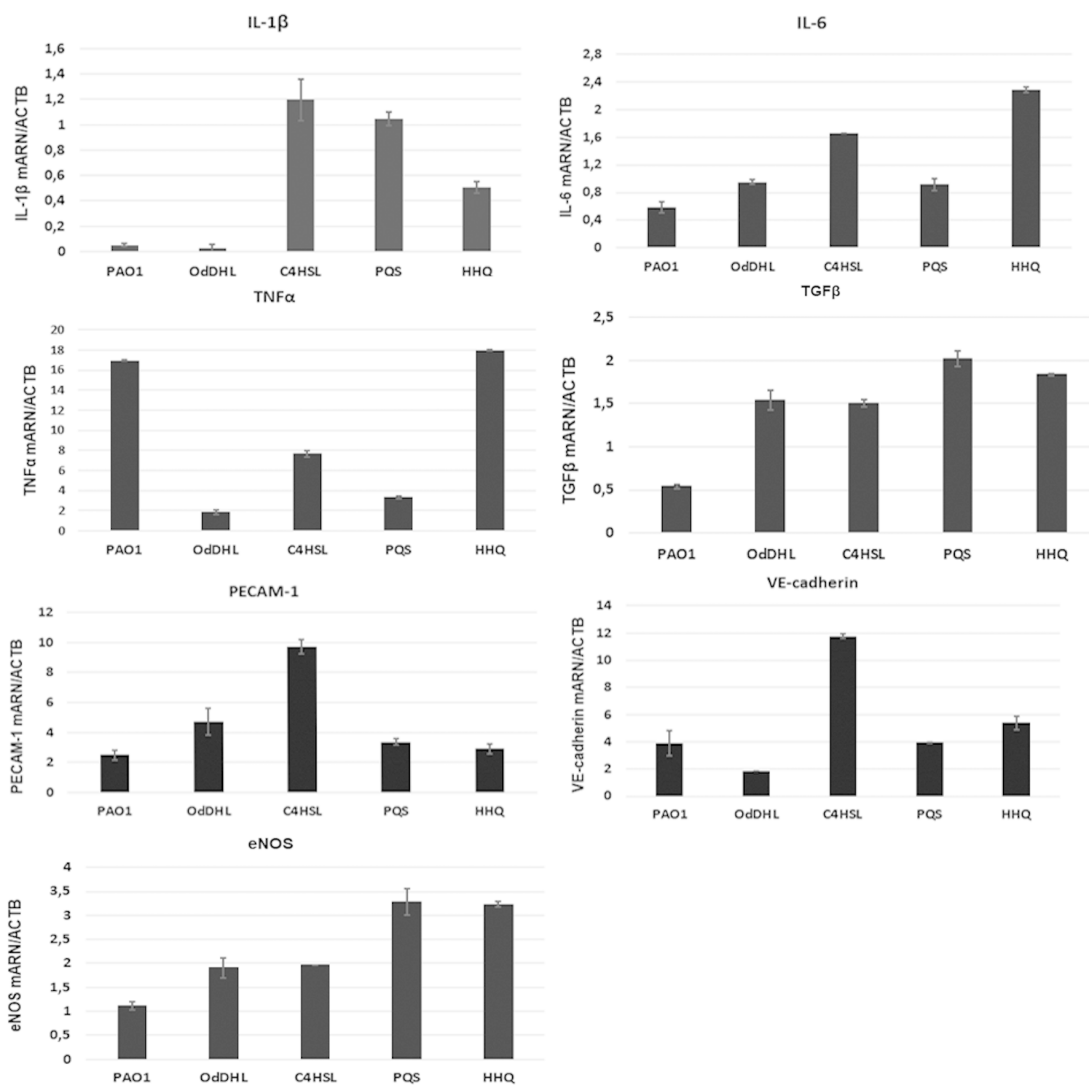
Expression levels (fold change) in qRT-PCR experiments for IL-1β, IL-6, TNFα, TGFβ, eNOS, VE-cadherin and PECAM-1 molecules performed on endothelial cells treated with PAO1 cultures and purified QSSMs (*P* < 0.05, based on ANOVA and Bonferroni post test of medians of 4 independent experiments performed in triplicate (*n* = 3)).

Another signaling molecule produced by *P. aeruginosa* has been shown to have immunomodulatory effects in the host cells [14,19]. PQS inhibits cellular proliferation without affecting IL-2 cytokine release when T cells are activated. PQS and OdDHL significantly reduce the ability of human peripheral mononuclear cells to respond to Con-A and to anti-CD3 and anti-CD28 antibodies. PQS does not affect cell viability while OdDHL inhibits cell proliferation and viability [20]. In addition, OdDHL inhibits the release of IL-2 and TNFα while PQS stimulates the release of these cytokines [21]. OdDHL can control PQS production, demonstrating that both molecules, while independent, with similar or different effects, act together [22]. The expression of adhesion molecules VE-cadherin and PECAM-1 was evaluated by qRT-PCR assay, and the obtained results sustained the flow cytometry data, suggesting the ability of QSSMs to modulate adhesion of host cells. An increased expression by 9 and 11 fold, respectively, was obtained when endothelial cells were treated with C4HSL (Figure 5). Migration of leukocytes from vessels into infected tissues involves interaction with endothelium through adhesion molecules. Adhesion molecules such as integrins (Cd11/Cd18) and ICAMs are usually involved in the inflammatory process during infection. *P. aeruginosa*-activated mast cells produce IL-1α and IL-1β, which stimulate the expression of ICAM-1 and E-selectin in endothelial cell, this process being required for transendothelial migration [23]. Furthermore Lins et al. showed 2010 that ICAM-1 expression is modulated by ExoU cytotoxin injected via the type III secretion system, being involved in endothelial damage and bacterial dissemination [24].

### Inflammatory markers

Proinflammatory cytokines and chemokines such as IL-1α, IL-1β, IL-6 and TNFα are produced by vascular cells such as endothelial cells and leucocytes within hours after *P. aeruginosa* infection. Flow cytometry assay showed elevated levels of IL-1β and IL-6. The level of IL-1β was increased after the stimulation with 20 µM QSSMs. The most significant IL-1β expression stimulation was observed in the sample treated with OdDHL (21.8% ± 0.09), as well C4HSL (21% ± 0.03), followed by HHQ (15.3% ± 0.3). PQS showed no effect in the production of IL-1β in endothelial cells (Figure 4). The expression of IL-6 was also increased after cellular treatment with OdDHL (55% ± 0.02), PQS (85.2% ± 0.015), HHQ (55% ± 0.02), while C4HSL showed no significant effect in the modulation of IL-6 expression (Figure 4). QRT-PCR results showed that the presence of C4HSL, PQS and HHQ molecules increased the expression level of IL-1β, while the PAO1 and OdDHL did not affect the level of IL-1β. IL-6 expression was slightly increased in the presence of all QSSM (0.5–2.3 fold), the differences being more significant for C4HSL and HHQ. The expression of TGFβ, eNOS was significantly increased in the presence of all QSSMs, by 1.5–2.0 fold (TGFβ) and 1.2–3.2 fold (eNOS), respectively, suggesting an activated phenotype of endothelial cells. The expression levels of adhesion molecules such as VE-cadherin and PECAM-1 were stimulated by the QSSMs, high levels being observed in the cells grown in the presence of C4HSL (Figure 5). Though there are numerous recent studies focusing on the influence of bacteria QSSMs on host cells, they are chiefly about the effects of AHL molecules, whereas the interactions between host cells and PQS have remained largely unknown. As far as we know, this is the first publication showing how both *P. aeruginosa*-derived AHL and PQS impact adhesion and inflammatory parameters of endothelial cells.

Thomas et al. in 2006 tested the ability of natural 3-oxo-C12-AHL and 4 synthetic analogues to modulate cytokine production in the host cells and the results demonstrated that these soluble mediators stimulate the production of TNFα and nitric oxide (NO) in equine and murine macrophages [25]. The mechanism of action of QSSMs leads to the activation of NFκB signaling cascade, resulting in the upregulation of pro-inflammatory cytokines IL-6, IL-8, and TNFα. However, our results demonstrated that TNFα expression is inhibited by all tested QSSMs, excepting HHQ (Figure 5). The expression of inflammatory molecules is tissue dependent and seems to be influenced by soluble virulence factors produce by *P. aeruginosa* during infection. Previous studies showed that most *P. aeruginosa* QSSMs increases the level of IL-6 in vitro. While HHQ is significantly stimulating IL-10 secretion by more than 20-fold, PQS has no significant effect on IL-10 secretion in mesenchymal stem cells [10]. Bacterial lipopolysaccharides (LPS) and phospholipase C (PLC) induces high levels of TNFα, IL-1β, IFNγ, MIP-1 and MIP-2 in lung cells, but they do not affect IL-18 levels [26]. In the tracheal epithelium LPS stimulates TLR2 and TLR4, while flagellin stimulates TLR5 expression. Alveolar epithelial cells respond to *P. aeruginosa* by releasing IL-1β, IL-6, IL-8, MIP-2 while alveolar macrophages are producing IL-1β and IL-18 [27].

## Conclusion

This is the first article about how *P. aeruginosa* derived QSSMs influence human endothelial cell adhesion and cytokine profile. Our findings demonstrate that the major pseudomonadal AHL and PQS auto-inducers differentially modulate bacteria adherence to inert and cellular substrata and biofilm formation, but also endothelial cells adhesion and pro-inflammatory cytokine expression. These observations may help in gaining insights into understanding host–pathogen interaction and communication and may have an impact on the development of new anti-QS therapeutic strategies to fight *P. aeruginosa* infections. Specifically, our results bring new information to help elucidating the mechanisms by which different QSSMs activate the host endothelial cells and promote epithelial lesions during *P. aeruginosa* infections.

## Experimental

Adherence assay to inert substrate and biofilm formation was assessed by the adapted microtiter method [28]. Overnight PAO1 cultures were grown with QSSMs in 96 multi-well plates containing Tryptic Soy Broth (TSB) for 24 h, 48 h and 72 h at 37 °C. After each period of time, the plates were subsequently emptied and washed three times with phosphate buffered saline (PBS). The adherent cells were then fixed with cold methanol, stained with an alkaline 1% crystal violet solution for 15 minutes, washed with distilled water and resuspended in a 33% acetic acid solution. The intensity of the suspension was spectrophotometrically assessed, the amount of adhered biomass being proportional to the absorbance value read at 492 nm.

### Adherence assay to cellular substrate

**Cell culture.** Endothelial cell line EA.hy926 was cultured in Dulbecco’s Modified Eagle’s Medium (DMEM, Sigma-Aldrich, St. Louis, MO, USA) containing 10% heat inactivated fetal bovine serum (FBS), 100 U/mL penicillin, 100 μg/mL streptomycin, and 50 μg/mL neomycin. Cell cultures were maintained at 37 °C in a humidified atmosphere containing 5% CO_2_ and 21% O_2_. To maintain optimal culture conditions, the medium was changed twice a week. Prior the infection with *P. aeruginosa* and adding QSSMs the endothelial cells were cultured in a medium without antibiotics. Endothelial cells were stimulated with *P. aeruginosa* PAO1 strain (control) and purifyed QSSMs (20µM) (OdDHL = *N*-(3-oxododecanoyl)-L-homoserine lactone; C4HSL = *N*-butyryl-L-homoserine lactone; PQS = 2-heptyl-3-hydroxy-4(1*H*)-quinolone; and HHQ = 2-heptyl-4-quinolone). For the adherence assay, Cravioto’s adapted method was used [29]. Briefly, the endothelial cells monolayers were washed with PBS (phosphate buffered saline) and 1 mL of fresh medium without antibiotics was added to each well. Suspensions of *P. aeruginosa* were obtained from bacterial mid-logarithmic phase cultures grown in nutrient broth adjusted to 10^7^ CFU/mL and 1 mL were used for the inoculation of each well in the presence of QSSMs. The inoculated plates were incubated for two hours at 37 °C. After incubation, endothelial cells were washed three times with PBS, fixed with cold methanol and stained with 5 µg/mL propidium iodide. The plates were washed, dried at room temperature and examined immediately with a fluorescent microscope (Eclipse TE300, with Digital Net Camera DN100, Nikon, Tokyo, Japan), by using the 100×, immersion oil objective.

The plates were examined microscopically to evaluate the adherence index and patterns. The adherence index was expressed as the ratio between the number of the eukaryotic cells with adhered bacteria and 100 eukaryotic cells counted on the microscopic field. The adherence patterns were defined as: localized adherence (LA) when tight clusters of microorganisms were noticed on the HeLa cell surface, aggregative adherence (AA) when a microbial stacked brick pattern characterizes the attachment, diffuse adherence (DA) when the bacteria adhered diffusely, covering the whole surface of the cell.

### QRT-PCR assay

The gene expression level of adhesion and inflammatory molecules in endothelial cells treated with PAO1 cultures and QSSMs, was assessed by qRT-PCR. Total cellular RNA was isolated using RNeasy Mini kit (Qiagen, Hilden, Germany) and reverse-transcription reaction was performed using M-MLV polymerase (Thermo Fisher Scientific, Massachusetts, USA). The mRNA levels were quantified by amplification of cDNA using a real-time thermocycler (Viia7 Applied Biosystems thermalcycler, Thermo Fisher Scientific, Massachusetts, USA) and TaqMan chemistry using the following assays: IL-1β (Hs01555410_m1), IL-6 (Hs00174131_m1), TNFα (Hs00174128_m1), TGFβ (Hs01086000_m1), eNOS (Hs01574665_m1), VE-cadherin (Hs00975233_m1), PECAM-1 (Hs01065279_m1), GAPDH (Hs03929097_g1). PCR reaction had the following schedule: denaturation step at 95 °C for two minutes, 40 cycles of denaturation at 95 °C, 15 seconds and annealing at 60 °C, 40 seconds. The genes expression level was normalized to GAPDH. The relative quantification was done using the comparative CT method and expressed as arbitrary units.

### Flow cytometry assay

Endothelial cells were stimulated with *P. aeruginosa* PAO1 strain and QSSMs (20 µM) for 20 hours and the expression of cell adhesion and inflammatory markers was assessed by flow cytometry (Gallios, Beckman-Coulter) using 1 × 10^5^ endothelial cells stained with fluorochrome-conjugated (FITC - fluorescein-isothiocyanate and PE - phycoerythrin) primary antibodies against IL-1β, IL-6, ICAM-1 PECAM-1, P-selectin, VE-cadherin (Beckman-Coulter). Endothelial cells were detached using trypsin (Sigma-Aldrich, USA) and washed in PBS. Cells were then incubated with the primary antibodies at room temperature in the dark for 30 min. Further, the cells were washed twice and centrifuged at 400*g*, 10 min, in PBS suplemented with 1% BSA. For negative controls, endothelial cells were stained with the corresponding isotype-matched IgG antibodies (Beckman-Coulter). Flow cytometry data were analyzed using the Gallios software (Beckman-Coulter).

### Statistical analysis

One way analysis of variance (ANOVA) was used to analyze the data (GraphPad software). Bonferroni post test was used when appropriate. *P* values < 0.05 were considered significant.
